# Modulatory Role of Vitamin E on Proton Pump (ATPase) Activity of Cadmium Chloride-Induced Testicular Damage in Wistar Rats

**DOI:** 10.1155/2021/4615384

**Published:** 2021-02-01

**Authors:** Olugbemi T. Olaniyan, Abdulfatai O. Ojewale, Olugbenga O. Eweoya, Adetola A. Adedoyin, Olamide A. Adesanya, Azeez O. Adeoye, Olatayo S. Okeniran

**Affiliations:** ^1^Laboratory for Reproductive Physiology and Developmental Programming, Department of Physiology, Edo University Iyamho, Edo State, Nigeria; ^2^Department of Anatomy, Kampala International University, Bushenyi, Uganda; ^3^Department of Anatomy, College of Health Sciences, University of Abuja, Abuja, Nigeria; ^4^Department of Anatomy, School of Medicine, University of Gambia, Banjul, Gambia; ^5^Department of Physiology, Bingham University Karu, Nasarawa State, Nigeria

## Abstract

Proton pumps are membrane-bound enzymes important in generating gradients that help in maintaining cellular ion homeostasis, cell membrane potential, water, and solute transport across the cell surface. This study investigated the modulatory role of vitamin E on proton pump activity and reproductive parameters in cadmium-induced testicular damage. Twenty (20) male Wistar rats weighing between 180 and 200 g were sorted into 4 groups of five rats each. Group I served as the control and was given normal saline orally, Group II rats were treated with a single dose of 2 mg/kg BW cadmium chloride (CdCl_2_) intraperitoneally, Group III rats were given 100 mg/kg BW of vitamin E orally, and Group IV rats were given 100 mg/kg BW of vitamin E orally for 30 days prior to intraperitoneal administration of single dose of 2 mg/kg BW of cadmium chloride. The rats were anaesthetized with diethyl ether, and blood samples were obtained for sex hormonal analysis; caudal epididymis was dissected for sperm count, motility, and viability, and the testis were homogenized for lipid peroxidation and proton pump (Na^+^/K^+^ ATPase, Ca^2+^ ATPase, and Mg^2+^ ATPase) activity. Proton pump activity was assayed spectrophotometrically using the Stewart method to determine the inorganic phosphate level. Histopathological changes of the testis were also studied. The group treated with CdCl_2_ showed a significant (*p* < 0.05) decrease in proton pump activity, sperm count, and motility and a significant (*p* < 0.05) increase in malondialdehyde level when compared with the control group. The CdCl_2_-treated group also showed decrease reproductive organ weights and hormonal levels and cause necrosis of spermatogonia lining the seminiferous tubules. Rats treated with vitamin E orally for 30 days prior to CdCl_2_ exposure showed improvement in proton pump activity, a significant (*p* < 0.05) increase in sperm parameters and luteinizing hormonal level, and a decrease in the lipid peroxidation level as compared with the CdCl_2_ group. This study showed that vitamin E ameliorated the toxic effect of CdCl_2_ on proton pump activity in the testes, hence improving testicular integrity, structures, and functions.

## 1. Introduction

Infertility is defined as the lack of ability of a noncontracepting couple to conceive after repeated and unprotected sexual intercourse for more than one year [1]. The male factor is solely responsible for infertility in approximately 20% of couples and a contributing factor in another 30-40% of couples; as such, the male factor is implicated in more than 50% of couples attempting to conceive [2]. Male factor infertility is seen as an alteration in sperm concentration, motility, or morphology in at least one sample of two sperm analyses, collected in the first and fourth weeks of analyses [3]. A reduction in male fertility potential may be due to congenital or acquired conditions such as urogenital abnormalities, varicocele, infections of the genital tract, genetic abnormalities, endocrine disturbances, testicular failure, immunologic problems, cancer, systemic diseases, altered lifestyle, and exposure to gonadotoxic factors such as cadmium, alcohol, and tobacco [4]. Humans are exposed to various types of environmental contaminants at different stages of their lifespan; the majority of which are harmful. Cadmium (Cd^2+^) is a toxic heavy metal occurring in the environment naturally and as a pollutant emanating from industrial and agricultural sources [5]. Cadmium has been found to produce a wide range of biochemical and physiological dysfunctions in humans and laboratory animals [6]. Exposure to cadmium can negatively affect the male reproductive system via degenerative changes in the testes, epididymis, and seminal vesicles [7, 8]. Exposure to cadmium leads to increased testicular lipid peroxidation, thereby impairing intracellular defenses leading to altered spermatogenesis [9]. Testicular oxidative stress is commonly induced under different normal or pathophysiological conditions, leading to male infertility [10]. Exposure to cadmium has an effect of a significant reduction in the enzymatic activities of superoxide dismutase, glutathione peroxidase, and catalase in testicular cells [9] and a depletion of many essential metal antioxidants including selenium in the body [11]. Cadmium also interferes with transport across cell membranes and epithelium and may therefore disturb the homeostasis and function of the cell. Na^+^/K^+^ ATPase is a membrane-associated enzyme that uses the energy from the hydrolysis of adenosine triphosphate (ATP) to transport 3 Na^+^ out of the cell in exchange for 2 K^+^ that is taken in [12]. In epithelial cells, Na^+^/K^+^ ATPase helps create the appropriate medium inside the seminiferous tubule lumen for the normal development of the spermatozoa [13] and in maintaining cell ion homeostasis, cellular luminal fluid, seminiferous and epididymal muscle contraction necessary for testicular function. Cadmium (Cd^2+^) exerts a potent inhibitory effect on Na^+^/K^+^ ATPase, and Mg^2+^ ATPase by binding avidly to vitamin E is a major lipid-soluble antioxidant located primarily within the phospholipid bilayer of cell membranes. Vitamin E is synthesized only by plants and, therefore, is found in plant products; the richest sources include vegetable oils and to a lesser extent, seeds, nuts, and cereal grains. It is particularly effective in preventing lipid peroxidation, a series of chemical reactions involving the oxidative deterioration of polyunsaturated fatty acids (PUFAs) [14]. Vitamin E, a nonenzymatic antioxidant, has been shown to offer protection against cadmium-induced free radical-mediated testicular damage [15–17]. Vitamin E interrupts the free radical chain reaction involved in lipid peroxidation and directly neutralizes superoxide anion, hydrogen peroxide, and hydroxyl radical. This study therefore is aimed at investigating the toxic effect of cadmium-chloride on proton pump activity in the testes and to evaluate the possible modulatory role of vitamin E on the proton pump activity and reproductive parameters of Wistar rats.

## 2. Materials and Methods

### 2.1. Sources of Chemicals, Reagents, and Kits

Cadmium chloride (Sigma Chemical Co., USA) and vitamin E (Cixon Pharma Co., India) were purchased from their local representatives in Nigeria. All other chemicals and reagents used in this study were of analytical grade.

### 2.2. Animal Source and Handling

Twenty (20) adult male Wistar rats weighing between 180 and 200 g obtained from the National Veterinary Research Institute, Vom, Jos, Nigeria, were used in this study. The animals were brought to the Department of Physiology, Bingham University animal house facility, where they were acclimatized for two weeks before the start of the experiment. They received food (grower's feed) and water *ad libitum* throughout the period of the experiment. The use and care of the animals and the experimental protocol were in strict compliance with the Institute of Laboratory Animals Research (ILAR) guidelines, 1996 [18].

### 2.3. Experimental Protocol

#### 2.3.1. Animal Grouping

The animals were randomly divided into four groups of five rats each as follows:

Group I: animals in this group were given normal saline orally for 30 days and served as controls

Group II: animals in this group were treated with a single dose of CdCl_2_ (2 mg/kg BW) intraperitoneally on day 30 of the experimental period

Group III: animals in this group were given vitamin E (100 mg/kg BW) orally for 30 days

Group IV: animals in this group were pretreated with vitamin E (100 mg/kg BW) for 30 days orally and then administered with a single dose of CdCl_2_(2 mg/kg BW) intraperitoneally

### 2.4. Sample Collection for Biochemical Analysis

In all groups, blood samples were collected retroorbitally; sera obtained were stored at 4°C for subsequent ELISA analysis for the determination of reproductive hormones. The rats were anaesthetized with diethyl ether, and the testes were collected, cleared of adherent tissue, and weighed using a sensitive weighing balance.

### 2.5. Semen Analysis

The left epididymis of each rat was used for the determination of epididymal sperm concentration using the Neubauer hemocytometer, while % sperm motility was determined as previously described by Sonmez et al. [19]. The epididymal sperm content was obtained by maceration of the tail of the epididymis on a dry, clean, and warm slide, mixing well with a drop of warm normal saline solution and immediately examined under the (10x) objective lens of a light microscope (Olympus). A drop of epididymal content of each rat was mixed with an equal drop of eosin-nigrosin stain prepared in accordance with Barth and Oko [20]. Thin films were made by spreading the stained content onto clean slides and quickly dried. Viable sperm remain colorless. One hundred sperm cells per rat were scored for determining the viability percent.

### 2.6. Assay of Lipid Peroxidation (Malondialdehyde)

Malondialdehyde levels were estimated by the method previously described by Evans [21]. This was measured as an indicator of lipid peroxidation and by extension reactive oxygen species. Testicular homogenates prepared was placed in microcentrifuge tubes and incubated with thiobarbituric acid (TBA). Following the incubation, samples were centrifuged (2000 rpm, 10 min) and the absorbance of the pink clear supernatant was measured at 532 nm in duplicate samples. Malondialdehyde bis(dimethyl acetate) was used as the external standard. The thiobabutric acid reactive material was expressed in terms of nanomoles of MDA/gm wet tissue.

### 2.7. Determination of Testicular and Epididymal Proton Pump (ATPase) Activities

The activities of sodium/potassium, calcium, and magnesium ATPase enzymes were assayed by the modified method previously described by Evans et al. [22] and Stewart et al. [23] and measured using a spectrophometry method to determine the inorganic phosphate in the testicular and epididymal tissue homogenates according to the method previously described by Kartha and Krishnamurthy [24].

### 2.8. Statistical Analysis

Data are presented as mean ± SEM. One-way ANOVA was performed with the Statistical Package for the Social Sciences (SPSS package version 16) to test the significance of differences between the results. The difference was considered significant at *p* < 0.05.

## 3. Results

### 3.1. Effect of Cadmium Chloride and Vitamin E on Body and Testicular Weights

The control group (I) gained weight over the thirty days of the experimental period, with the mean body weight increasing by 32.4 g after 30days (Table 1). In contrast, the cadmium chloride group (II) lost an average of 12.8 g after 30 days (*p* < 0.05). Treatment with vitamin E resulted in a significant weight gain by 46 g after 30 days at levels approaching the control group, and treatment with vitamin E for 30 days orally and then administration with a single dose of cadmium chloride (2 mg/kg BW) resulted in weight gain (25.8 g) at levels approaching the control group (Groups II and III versus Groups I and IV). Mean testicular weight in the cadmium chloride group significantly (*p* < 0.05) decreased as compared to that of the control group, while treatment with vitamin E for 30 days orally and then administration with a single dose of cadmium chloride (2 mg/kg BW) decreased compared to treatment with vitamin E for 30 days orally increased significantly (*p* < 0.05).

### 3.2. Effect of Cadmium Chloride and Vitamin E on Lipid Peroxidation, Proton Pump Activities, and Semen Analysis of Male Wistar Rats

The group treated with vitamin E orally for 30 days prior to cadmium chloride administration (Table 2) showed a significant (*p* < 0.05) decrease in the lipid peroxidation level compared to the cadmium chloride group while the group treated with cadmium chloride showed a significant (*p* < 0.05) increase in lipid peroxidation level compared to the control group. Treatment with vitamin E orally for 30days prior to cadmium chloride administration intraperitoneally showed a significant (*p* < 0.05) increase in proton pump activity, sperm count, and motile sperm cells and a decrease in nonmotile sperm cells when compared with the cadmium chloride group (Table 2). This result (Table 2) showed that vitamin E is able to modulate the cadmium chloride-induced testicular damage from proton pump activity by regulating the fluid/lipid milieu of the testicular membrane which in turn maintained the membrane bound enzymes (ATPase).

### 3.3. Effect of Cadmium Chloride and Vitamin E on Hormonal Levels in Male Wistar Rats

The cadmium chloride group (Table 3) showed a decrease in serum luteinizing hormone, follicle-stimulating hormone, and testosterone levels when compared with the control group, while treatment with vitamin E for 30 days orally only and treatment with vitamin E for 30 days orally and then administration with a single dose of cadmium chloride (2 mg/kg BW) showed an increase in serum luteinizing hormone, follicle-stimulating hormone and testosterone levels when compared with the cadmium chloride group while the group treated with vitamin E orally for 30 days prior to cadmium chloride administration (Table 3) showed a significant (*p* < 0.05) increase in serum LH level and a significant (*p* < 0.05) decrease in FSH and testosterone levels. Inhibition of serum levels of LH, FSH, and testosterone by cadmium chloride resulted in altered spermatogenesis evident by a decrease in sperm count shown by the cadmium chloride group (Table 3). This abnormal endocrine response is as a result of direct destruction of the testes (Leydig and Sertoli cells), which are also endocrine organs and the hypothalamic pituitary testicular axis.

### 3.4. Histopathology of Testis

**Figure 1:**
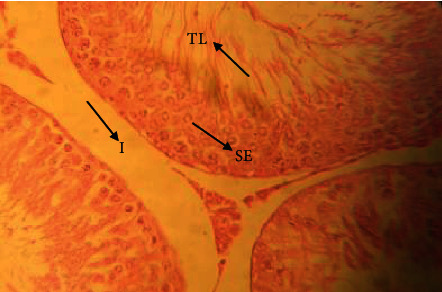
Histological appearance of testis in the control group. Showing seminiferous epithelium (SE) with normal spermatogonia, seminiferous tubule lumen (TL) with spermatozoa, and interstitial spaces (I) with no pathological damage (H&E, ×40 magnification).

**Figure 2:**
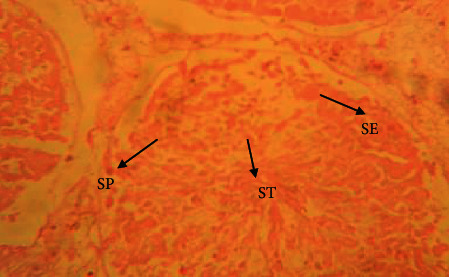
Histological appearance of the testis in the group treated with a single dose of 2 mg/kg BW of cadmium chloride. Seminiferous epithelium (SE) with degeneration of seminiferous tubule (ST), desquamation of germ cells, and necrosis of spermatogonial cells (SP) (H&E, ×40 magnification).

**Figure 3:**
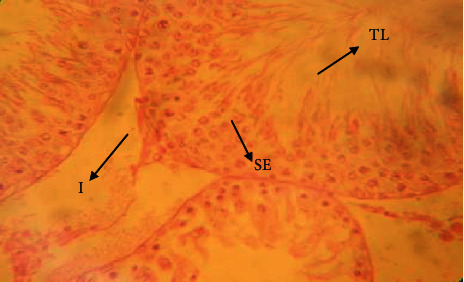
Histological appearance of the testis in the group treated with 100 mg/kg BW vitamin E showing seminiferous epithelium (SE) with spermatogonia, seminiferous tubule lumen (TL) with spermatozoa, and interstitial space (I) with no pathological damage (H&E, ×40 magnification).

**Figure 4:**
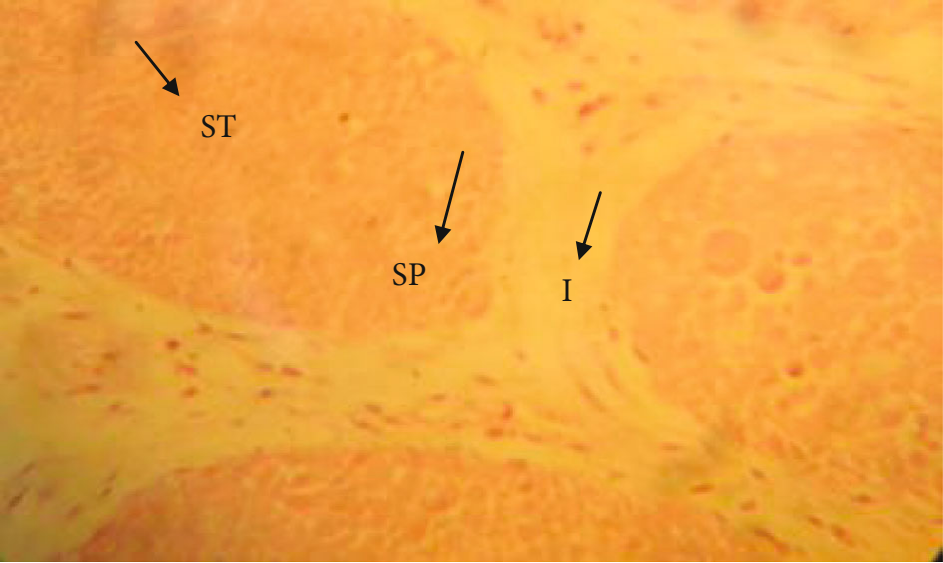
Histological appearance of the testis in the group treated with 100 mg/kg BW vitamin E orally prior to single-dose administration of 2 mg/kg BW cadmium chloride intraperitoneally. Showing regeneration of spermatogonial cells (SP), interstitial space I, and seminiferous tubule ST (H&E, ×40 magnification).

The testis of rats in the control group (Figure 1) exerted different stages in seminiferous elements comprising germ cells and interstitial cells, which are normal in appearance compared to rats in the cadmium chloride group. Cadmium exposure to the testes (Figure 2) showed atrophy and degeneration of seminiferous tubules. Seminiferous tubules contained a damaged germinal layer and disorganization of the cellular layers. Testes in the vitamin E (Figure 3) group revealed well-developed circular or elliptical seminiferous tubules containing spermatozoa, while the group treated with vitamin E orally for 30 days prior to cadmium chloride administration (Figure 4) showed regeneration of spermatogonial cell lines in the testis.

## 4. Discussion

In this study, the cadmium chloride group (Table 2) showed an increase in lipid peroxidation level marked by an increase in malondialdehyde level in the testicular tissue when compared with other groups which is in agreement with the study of Rekha et al. [15]. Cadmium chloride caused a decrease in testicular weight; this could be as a result of macromolecular degradation incurred by reactive oxygen species and damage to the testicular membrane leading to destruction of most of the germ cells in the testes thereby reducing testicular weight [15, 25]. The group treated with vitamin E orally for 30 days prior to cadmium chloride administration showed an increase in testicular weight and a significant (*p* < 0.05) decrease in lipid peroxidation level. Studies have shown that within two weeks, cadmium chloride would have affected the detrimental effect on spermatogenesis; thus, the treatment of vitamin E for 30 days for the antioxidant protection [15, 26]. This shows that vitamin E protected the testicular membrane from cadmium chloride-induced testicular damage, which is in agreement with the report of Rekha et al. [15]. Testicular lipid peroxidation alters the lipid milieu, structural and functional integrity, and the activities of various membrane-bound enzymes in the testicular membrane [26–28]. Membrane-bound enzymes such as ATPases generate a gradient that is essential in maintaining cell ion homeostasis, cell membrane resting potential, transport of a variety of solutes and water across the cell surface, the excitability of muscle and neuronal cells, and the Na^+^-coupled secondary transport of H^+^, Ca^2+^, glucose, amino acids, and neurotransmitters across the plasma membrane [12]. Lipid peroxidation has been shown to alter Na^+^/K^+^ ATPase function by modification at specific sites in a selective manner [27].

From the result obtained (Table 2), there was a significant (*p* < 0.05) decrease in proton pump activity in the cadmium chloride-treated group as compared with control. ATPases (Na^+^/K^+^ATPase, Mg^2+^ ATPase, and Ca^2+^ ATPase) are very sensitive to the presence of metal ions such as cadmium and lead [29]; cadmium ion (Cd^2+^) competes with ATPase cofactors (Na^+^, K^+^, Mg^2+^, and Ca^2+^) and form complexes with its functional group by nonspecific binding with sulfhydryl groups of cysteine residues leading to the loss of protein-SH groups, which is a consequence of lipid peroxidative damage [27]. Na^+^/K^+^ ATPase helps create the appropriate medium in the seminiferous tubule lumen for normal development of the spermatozoa [13]. Inhibition of proton pump activity could result in alkalinisation of the luminal fluid of the seminiferous tubule and epididymis; cadmium causes alkalinisation of fluid in the seminiferous tubule and epididymis in adult male rats [30]. Impaired acidification could result to instability in the resting membrane potential of germ cells, deficient sperm maturation, and motility leading to male infertility [31]. This can be related to the decrease in viable and motile sperm cells, an increase in nonviable and nonmotile sperm cells, and a significant decrease (*p* < 0.05) in sperm count associated with the cadmium-treated group when compared with the vitamin E and control groups. This is because the transmembrane distribution of electrical charges, resulting from the uneven Na^+^ and K^+^ transport activity of the Na^+^/K^+^-ATPase, contributes to maintain the resting membrane potential that provides cells with the excitability required for their movement, contraction of myosin and actin filaments on the peritubular cells of the seminiferous tubule, and transmission of impulses [32]. Treatment with vitamin E orally for 30 days prior to cadmium chloride administration intraperitoneally showed a significant (*p* < 0.05) increase in proton pump activity, sperm count, an dmotile sperm cells and a decrease in nonmotile sperm cells when compared with cadmium-treated group (Table 3). Follicle-stimulating and luteinizing hormones secreted from the anterior pituitary stimulate the interstitial cells of Leydig to secrete testosterone, which regulates testicular functions such as growth and division of testicular germinal cells [33]. In accordance with the results obtained from this study, there are reports indicating that cadmium chloride reduces serum hormonal levels of follicle-stimulating hormone and luteinizing hormone, which induce the signals for the synthesis of testosterone [16, 34]. An inhibition of these signals results in a reduction in serum testosterone levels, which leads to reproductive dysfunction, cell death, and apoptosis by cadmium.

## 5. Conclusion

This study showed that vitamin E ameliorated the toxic effect of cadmium on the proton pump activity (Na^+^/K^+^ ATPase, Mg^2+^ ATPase, and Ca^2+^ ATPase) in the testis. Vitamin E in the diet is encouraged as it is effective in neutralizing reactive oxygen species generated from cadmium and modulating the activity of the membrane-bound enzymes necessary for the maintenance of testicular function and integrity. This will therefore contribute to improving the fertility of among males who are exposed to heavy metals.

## Figures and Tables

**Table 1:** Effect of cadmium chloride and vitamin E on body and testicular weights.

Groups	Initial body weight (g)	Final body weight (g)	Gain in body weight (g)	Testicular wt (g)
I	181.0 ± 9.8	213.4 ± 18.4	32.4	0.96 ± 0.1
II	184.0 ± 16.0	196.8 ± 13.8^∗^	12.8	0.50 ± 0.1^∗^
III	186.0 ± 14.7	232.0 ± 13.2	46.0	1.00 ± 0.1
IV	180.0 ± 7.6	205.8 ± 15.7	25.8	0.55 ± 0.1

**Table 2:** Effect of cadmium chloride and vitamin E on semen analysis of male Wistar rats.

Parameters	Group			
	I	II	III	IV
Sperm count (×10^6^/ml)	66.6 ± 1.4	14.0 ± 2.8	85.0 ± 3.5^∗^^+^	33.2 ± 2.1^∗^^+^
Motility (%)	84.0 ± 4.0	18.0 ± 5.8	80.0 ± 6.3^+^	58.0 ± 7.4^∗^^+^
Viability (%)	76.0 ± 2.5	74.0 ± 5.1	82.0 ± 2.0	80.0 ± 0.0
MDA (nmol/mg^−1^protein)	2.97 ± 2.6	8.3 ± 1.7	1.3 ± 0.5^+^	4.2 ± 1.3^∗^^+^
Na^+^/K^+^ ATPase (P_1_*μ*mol/mgPr^−^/hr)	2.4 ± 0.7	1.1 ± 0.3	2.4 ± 0.4	2.0 ± 1.2^+^
Ca^2+^ ATPase (P_1_*μ*mol/mgPr^−^/hr)	2.8 ± 0.6	1.6 ± 1.2	3.4 ± 1.4^+^	4.0 ± 1.5^+^
Mg^2+^ ATPase (P_1_*μ*mol/mgPr^−^/hr)	2.7 ± 0.3	2.4 ± 0.8	2.5 ± 0.1	3.9 ± 0.4^+^

Values are represented as mean ± standard error of the mean, *n* = 5. ^∗^When compared with the control group, *p* < 0.05; ^+^when compared with the cadmium chloride group at *p* < 0.05.

**Table 3:** Effect of cadmium chloride and vitamin E on hormonal levels in male Wistar rats.

Group	I	II	III	IV
FSH (miU/ml)	3.17 ± 0.10	0.15 ± 0.01^∗^	0.81 ± 0.06	0.26 ± 0.04^+^
LH (miU/ml)	2.26 ± 0.15	0.14 ± 0.01^∗^	0.29 ± 0.02	0.70 ± 0.01^+^
Testosterone (ng/ml)	4.06 ± 0.09	0.18 ± 0.13^∗^	1.52 ± 0.61^+^	0.88 ± 0.02^+^

Values are represented as mean ± standard error of the mean, *N* = 5. ^∗^When compared with the control group at *p* < 0.05, ^+^when compared with the cadmium chloride group at *p* < 0.05.

## Data Availability

The datasets generated during the analyses used to support our findings of this study are available from the first and corresponding authors on reasonable request.
